# Transcriptome Analysis Reveals POD as an Important Indicator for Assessing Low-Temperature Tolerance in Maize Radicles during Germination

**DOI:** 10.3390/plants13101362

**Published:** 2024-05-14

**Authors:** Yifei Zhang, Jiayu Li, Weiqing Li, Xinhan Gao, Xiangru Xu, Chunyu Zhang, Song Yu, Yi Dou, Wenqi Luo, Lihe Yu

**Affiliations:** 1 College of Agriculture, Heilongjiang Bayi Agricultural University, Daqing 163319, China; byndljy@163.com (J.L.); lwq_ljy@163.com (W.L.); 13199155147@163.com (X.G.); 18822901166@163.com (X.X.); 13684592065@163.com (C.Z.); yusong@byau.edu.cn (S.Y.); bynddy@163.com (Y.D.); byndlwq@163.com (W.L.); 2 Heilongjiang Provincial Key Laboratory of Modern Agricultural Cultivation and Crop Germplasm Improvement, Daqing 163319, China; 3 Key Laboratory of Low-Carbon Green Agriculture in Northeastern China, Ministry of Agriculture and Rural Affairs, Daqing 163319, China

**Keywords:** transcriptome, seed germination, TS, maize radicle, differentially expressed genes, phenylpropanoid biosynthesis

## Abstract

Low-temperature stress (TS) limits maize (*Zea mays* L.) seed germination and agricultural production. Exposure to TS during germination inhibits radicle growth, triggering seedling emergence disorders. Here, we aimed to analyse the changes in gene expression in the radicles of maize seeds under TS by comparing Demeiya1 (DMY1) and Zhengdan958 (ZD958) (the main Northeast China cultivars) and exposing them to two temperatures: 15 °C (control) and 5 °C (TS). TS markedly decreased radicle growth as well as fresh and dry weights while increasing proline and malondialdehyde contents in both test varieties. Under TS treatment, the expression levels of 5301 and 4894 genes were significantly different in the radicles of DMY1 and ZD958, respectively, and 3005 differentially expressed genes coexisted in the radicles of both varieties. The phenylpropanoid biosynthesis pathway was implicated within the response to TS in maize radicles, and peroxidase may be an important indicator for assessing low-temperature tolerance during maize germination. Peroxidase-encoding genes could be important candidate genes for promoting low-temperature resistance in maize germinating radicles. We believe that this study enhances the knowledge of mechanisms of response and adaptation of the maize seed germination process to TS and provides a theoretical basis for efficiently assessing maize seed low-temperature tolerance and improving maize adversity germination performance.

## 1. Introduction

Several complex physiological processes participate in seed germination, including water uptake, storage material degradation, seed respiration, gene expression, mitochondrial repair, and proliferation [1]. Environmental and intrinsic factors heavily influence seed germination at the beginning of the plant life cycle [2]. Temperature is an important external condition that affects seed germination; at optimum temperature, the seed rapidly absorbs water to soften the seed coat, enabling the radicle and the germ to emerge from the seed coat and begin the germination process [3]. However, under low ambient soil temperatures, the rate of seed water uptake slows and becomes more unfavourable for the growth and development of embryos [4]. In the context of global climate change, the frequency and intensity of weather extremes are increasing, which significantly inhibits seed germination and plant growth [5,6]. However, low temperature is considered to be among the primary abiotic stresses for growth, development, and spatial distribution of plants, induction of the production of many reactive oxygen species (ROS) in plant cells (such as O_2_^-^ and H_2_O_2_), and triggering lipid peroxidation in the membranes. Simultaneously, it slows down the repair of the mitochondrial membrane and reduces the activity of enzymes involved in respiration, leading to prolonged germination time and reduced seedling emergence [7,8], and even severely restricts crop yield. Therefore, an in-depth and systematic investigation of the response and adaptation mechanisms of seeds to low-temperature stress (TS) during germination is essential to improve crop yields.

Maize (*Zea mays* L.) is one of the most important crop species globally and the main source of raw materials for animal feed and industry [9]. Grain production is an important commodity in China. The maize-sown area in Northeast China accounts for over 30% of the national total sown area of maize, and the total output accounts for 29% of the national total. Thus, maize occupies an important position in China’s food security guarantee system and the development of the agricultural economy [10]. Although the higher ambient temperature is generally favourable to maize production in the context of global climate change [11], climate warming still affects winter, which spans half of the year. Further, the warming of the maize growing season is not substantial, and the frequency of severe low-temperature and cold damage in Northeast China (particularly in the colder regions at high latitudes: Heilongjiang Province; Northern Jilin Province) significantly increases with the northward and eastward extension of the planting areas of maize varieties with different maturity types. The early and late growth stages of maize growth and development are the main stages of low-temperature occurrence [12,13]. Additionally, maize sowing in the region occurs during the spring and primarily uses the direct sowing technology. Low-temperature environments post sowing often present challenges to germination and seedling formation, resulting in reduced and delayed seedling emergence, reduced seedling formation rate, and an increase in the proportion of weak seedlings. In extreme cases, seeds may completely lose their ability to germinate, resulting in a substantial lack of seedlings over a large area [14,15]. Obtaining a high-quality maize population is a challenging aspect of the mechanisation and efficient production of maize in the region, restricting improvement in efficiency and the achievement of sustainable development in the regional maize industry.

Plants under TS undergo a range of morphological and physiological changes [16,17,18]. Physiological changes include the hardening of cell membranes, ROS accumulation, destabilisation of proteins, and metabolic disturbances [19]. Morphological changes include delayed germination, reduced germination rate, and impaired seedling growth [20]. Plants usually survive a brief exposure to low temperatures by initiating a series of defence mechanisms, whereas prolonged exposure can result in necrosis or death [21,22]. To overcome the detrimental effects of exposure to cold temperature (which do not reach freezing levels), plants have developed several pathways to counterbalance stress-induced damaging effects, evolving elaborate mechanisms for the biosynthesis of secondary metabolites such as terpenoids, phenylpropanoids, and alkaloids [23]. One of the major pathways in plants for the synthesis of most of the secondary metabolites is the phenylpropanoid pathway [24]. Phenylpropanoid compounds are a group of different secondary metabolites of plants derived from the phenylalanine carbon skeleton [25]. Phenylalanine ammonia-lyase (PAL) is the first rate-limiting enzyme in phenylpropanoid biosynthesis pathway, which is present in almost all plant cells and is involved in plant cell defence reactions and responses to biotic and abiotic stresses [26]. Trans-cinnamate 4-monooxygenase (C4H) catalyses the formation of *p*-coumaric acid from cinnamic acid, and it is the second critical enzyme in the phenylpropanoid pathway; 4-coumarate-CoA ligase (4CL) acetylates coumaroyl acids to produce *p*-coumaroyl coenzyme A esters [27]. Stress response is thought to induce structural gene expression of phenylpropanoid metabolic pathways, and changes in ambient temperature usually alter the content and type of phenylpropanoid compounds in plants [28]. These changes are the main intrinsic regulatory pathways affecting cold tolerance in plants [24,29]. Radicle growth, which is a main component of seed embryo germination, is considerably affected by low temperature and water stress during seed germination and seedling emergence. Radicle length is widely used as a key indicator for evaluating abiotic stress tolerance to low temperature and drought in crops such as maize [30], rice [31], and cotton [32,33]. However, the effects of TS on maize seed germination and seedling stage have been extensively studied in terms of phenotype [34], physiology and biochemistry [35], transcriptome [36], and metabolome [37]. Transcriptome analyses have not been conducted on maize radicles under TS during seed germination.

In the present research, transcriptome sequencing technology was used to investigate the effects of TS on gene expression in the radicle at the germination stage of two genotypes of maize varieties, Demeiya 1 (DMY1) and Zhengdan 958 (ZD958). These varieties are mainly planted in the colder regions at high latitudes in northeastern China, and they annotate the functions of the differentially expressed genes (DEGs) in response to TS. Key metabolic pathways are revealed to be involved in the response of radicles to TS. The results from this study deepen the understanding of the molecular physiological of radicle adaptation during maize seed germination to TS and lay a theoretical foundation for efficient evaluation of low-temperature tolerance during the germination period of maize in the cold regions and the continuous improvement of the low-temperature adaptation ability in the future.

## 2. Materials and Methods

### 2.1. Test Varieties and Experiment Design

We selected the main maize varieties, Demeiya1 (DMY1, flint maize) and Zhengdan958 (ZD958, dent maize), which are cultivated in northeastern China, at high latitudes and in cold regions. For each variety, uniform and consistent seeds were selected (100-seed weights of 27 and 40 g for DMY1 and ZD958, respectively), and all seeds were sequentially sterilised in 1% NaClO solution for 20 min, surface sterilised for 1 min in 75% (*v*/*v*) ethanol solution, and rinsed repeatedly with sterile water six times before the experiment was implemented. Surface-sterilised seeds were then individually placed in a cardboard germination box (length/width/height ratio 14 cm/14 cm/6 cm) containing 10 mL of sterile water and placed in an artificially operated climatological chamber with a temperature of 15 ± 0.5 °C (DRX-330E, Ningbo Dongnan Instrument Co., Ningbo, China) (50% ± 5% relative humidity) for dark culturing. Germinated seeds with uniform radicle lengths (1.0–1.2 cm) were selected after 72 h of germination for the following treatments: (1) Control treatment (CT) under normal culture conditions: germinated maize seeds continued to be incubated at 15 ± 0.5 °C for 48 h in the dark. The control treatments for DMY1 and ZD958 varieties were denoted as DCT and ZCT, respectively. (2) TS treatment (TS): germinated maize seeds were transferred to a dark culture for 48 h at 5 ± 0.5 °C. The TS treatments for DMY1 and ZD958 varieties were DTS and ZTS, respectively.

### 2.2. Measuring Relevant Phenotypic Indicators

The maize seeds (10 seeds) in the germination box were kept at 15 °C for 72 h for germination and were transferred to another germination box. The initial radicle lengths were simultaneously measured and recorded as L_1_. Subsequently, after incubation continued at 15 °C (CT) or 5 °C (TS) for 48 h, the radicle lengths of the germinated seeds in the germination box were measured and recorded as L_2_; radicle growth (%) = (L_2_ − L_1_)/L_1_ × 100%. The average value of the radicle lengths of 10 maize seeds in each germination box was taken as one replicate, and four replicates were performed for each treatment.

For fresh and dry weight (DW) determination, 10 germinated maize seed radicles in each germination box of different maize varieties were collected after normal incubation and TS treatments, and the total fresh weight (FW) of 10 maize seed radicles in each germination box was measured using a Presica LS120A (Sartorius AG, Göttingen, Germany) balance. The samples were dried at 80 °C to a constant weight (DW) using an electric thermostatic drying oven (ED115, BINDER Environmental Testing Equipment Co., Ltd., Tuttlingen, Germany). The weight of each sample was then measured. The total FW and DW of the radicles of 10 maize seeds in each germination box were replicated, and four replicates were conducted for each treatment.

### 2.3. Peroxidase (POD), Malondialdehyde (MDA), and Proline (Pro) Measurements

Maize radicles were collected for physiological index determination under control and low-temperature conditions. POD activity was measured using the method developed by Tan et al. [38]. Pro content was determined based on the method developed by Bates et al. [39]. MDA content was determined using the method developed by Heath and Packer [40].

### 2.4. Sample Preparation and RNA Sequencing

Transcriptome analyses were performed on germinated maize seed radicles under normal culture and TS treatment conditions with three biological replicates per variety and treatment, and each biological replicate involved 80 maize seed radicles (approximately 1.0 g), resulting in 12 samples. Following the manufacturer’s instructions, total plant RNA was extracted using the RNAprep Pure Plant Kit (Tiangen Biotech Co., Ltd., Beijing, China), and further cDNA library construction of all test samples was completed at Biomarker Technologies (Beijing, China). Finally, cDNA sequencing was performed on the library using the Illumina HiSeq 4000 platform (www.biomarker.com.cn).

### 2.5. Filtering of Sequencing Data and Quantification of Gene Expression

Clean reads were obtained from raw data by removing data that contained adapters and low-quality reads. Clean reads were subsequently calculated for the Q20 and Q30 values, GC content, and level of sequence repeats. To use them for further analysis, HISAT2 was used to map all the filtered clean reads to maize reference genome (B73_RefGen_v4) [41]. Comparison results were assembled using string ties [42]. The gene expression levels of all samples were quantified using the number of Fragments Per Kilobase Per Million Mapped Reads (FPKM) calculated based on the gene length and the count of reads mapped to it [43].

### 2.6. Analysis of Fluorescence Quantitative PCR in Real Time

To examine the reproducibility and accuracy of DEGs based on transcriptome sequencing, six DEGs were randomly selected for qPCR validation. Total RNA was extracted from maize seed radicle samples (CT or TS) using TRIzol^®^ reagent (Invitrogen, Carlsbad, CA, USA). To determine RNA quality, a NanoDrop spectrophotometer (Thermo Fisher Scientific, Waltham, MA, USA) was used with a 1% agarose gel. Primers targeting these six genes were designed using Primer Premier 5.0 with *ZmActin* [44] as the internal reference gene (Table S1). A CFX96 real-time fluorescent quantitative PCR detection system (Bio-Rad Laboratories Inc., Hercules, CA, USA) was used. The PCR cycling conditions included 60 s of initial denaturation at 95 °C, followed by 15 s of denaturation at 95 °C and 30 s of annealing at 55 °C for a total of 39 cycles. Three biological replicate samples were included in each treatment (each biological replicate contained three technical replicates), and gene expression levels were calculated according to the 2^−∆∆Ct^ method [45].

### 2.7. Differentially Expressed Gene Analysis and Functional Enrichment Analysis

To identify DEGs in different treatments, differential expression analysis between groups was conducted using DESeq2. For differential expression analysis of two samples under TS, |log2 fold change (FC)| ≥ 1 and a false discovery rate (FDR) < 0.01 were employed as screening criteria. The DEGs were then analysed for their corresponding Gene Ontology (GO) terms and metabolic pathways using the GO and the Kyoto Encyclopaedia of Genes and Genomes (KEGG). The DEGs were analysed for enrichment through GO and KEGG pathways using the Cluster Profiler 4.4.4 [46]. The q-value of < 0.05 was used as the criterion to screen GO and KEGG enrichment pathways.

### 2.8. Metabolite Determination and Gene Homologous Sequence Comparison

The samples from different treatments were collected and sent to Biomarker Technologies, Beijing, China for the determination of relevant metabolites using liquid chromatography/tandem mass spectrometry (LC-MS/MS). First, 0.05 g of the sample was added to 1 mL of extraction solution (methanol/acetonitrile/water = 2:2:1, internal standard concentration 20 mg/L). The sample was homogenised in a grinder and sonicated in ice water for 10 min. Thereafter, it was allowed to stand at −20 °C for 1 h and centrifuged at 12,000× *g* for 15 min at 4 °C. Then, 500 µL of the supernatant was transferred to a new 1.5 mL tube and dried, and 160 µL of extraction solution (acetonitrile/water = 1:1) was added. The solution was vortexed for 30 s and sonicated in an ice bath for 10 min, followed by centrifugation at 12,000× *g* for 15 min at 4 °C to obtain the supernatant. We removed 120 μL of supernatant in a 2 mL injection vial and mixed 10 μL of each sample into a quality control (QC) sample for testing. LC-MS/MS analyses were performed on an ACQUITY UPLC I-Class PLUS system (Waters Corporation, Milford, MA, USA) with a Xevo G2-XS QTof system (Waters Corporation, Milford, MA, USA). Samples were analysed in positive and negative ion modes. MS raw data were collected using MassLynx software (version 4.2, Waters Corp., Milford, CT, USA) and processed by Progenesis QI 4.0 (Waters Corp., Milford, CT, USA). Genes related to the phenylpropanoid biosynthesis pathway were aligned for homologous sequences using the BLASTp function on the NCBI (https://www.ncbi.nlm.nih.gov/) website.

### 2.9. Statistical Analyses

Four replicates were performed for each experimental treatment, except transcriptome sequencing, and values were calculated as the mean ± standard deviation (SD) of the four replicates. Phenotypic and physiological index data were analysed employing analysis of variance using IBM SPSS Statistics for Windows version 19.0 (IBM Corporation, Armonk, NY, USA). Significant differences were compared between treatments with the new Duncan’s multiple range test, with *p* < 0.05 indicating a significant difference between treatment groups.

## 3. Results

### 3.1. Effect of TS on the Growth and Physiological Characteristics of the Radicles of Maize Seeds

**Figure 1:**
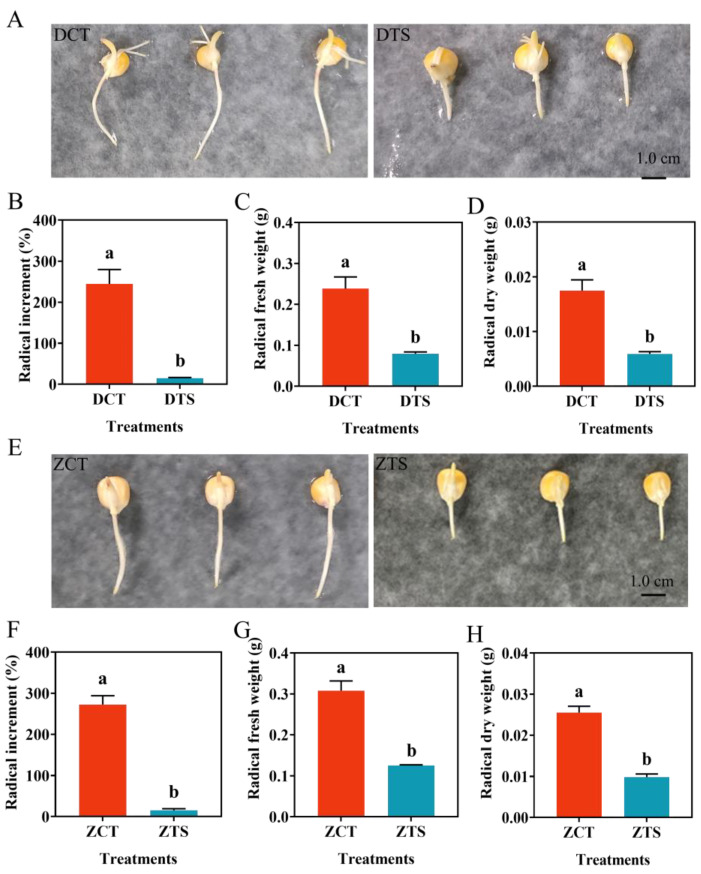
Phenotypic changes induced by low temperatures on radicle growth in maize seeds. (**A**,**E**) are observations and photographs of DMY1 and ZD958 germinated seeds after 48 h of incubation under CT or TS conditions, respectively; (**B**,**F**) represent the percentage of radicle length of germinated seeds of the two varieties determined after 48 h of incubation under CT or TS conditions, respectively; (**C**,**G**) are measurements of the fresh radicle weight of the germinated seeds of the two varieties after 48 h of incubation under CT or TS conditions, respectively; (**D**,**H**) are measurements of the dry radicle weight of the germinated seeds of the two varieties after 48 h of incubation under CT or TS conditions, respectively. CT: control treatment; DCT: control treatment of DMY1; DTS: low-temperature treatment of DMY1; TS: low-temperature stress; ZCT: control treatment of ZD958; ZTS: low-temperature treatment of ZD958. Data represent the mean ± standard error of four replicates. Means with different letters indicate significant differences between the different treatments (*p* < 0.05, Duncan’s multiple range test).

The changes in the radicle growth, FW, and DW of DMY1 and ZD958 after low-temperature treatment were examined to determine the effects of TS on radicle growth during maize seed germination (Figure 1). In terms of changes in radicle length, TS treatment significantly (*p* < 0.05) inhibited radicle elongation of DMY1 (Figure 1A) and ZD958 (Figure 1E) compared to normal culture conditions. The radicle length increased by 244.45% and 14.47% after 48 h of DCT and DTS treatments, respectively, for DMY1, while for ZD958, the radicle length increased by 271.15% and 15.34% after 48 h of ZCT and ZTS treatments, respectively. Additionally, the FW of DMY1 and ZD958 radicles decreased by 66.67% and 58.06%, respectively, compared to normal culture conditions, and the DW of radicles decreased by 66.08% and 61.45%, respectively, at a significant level (*p* < 0.05) after TS treatment. Based on these results, TS had a significant adverse effect on radicle growth in both maize varieties tested in this study.

**Figure 2:**
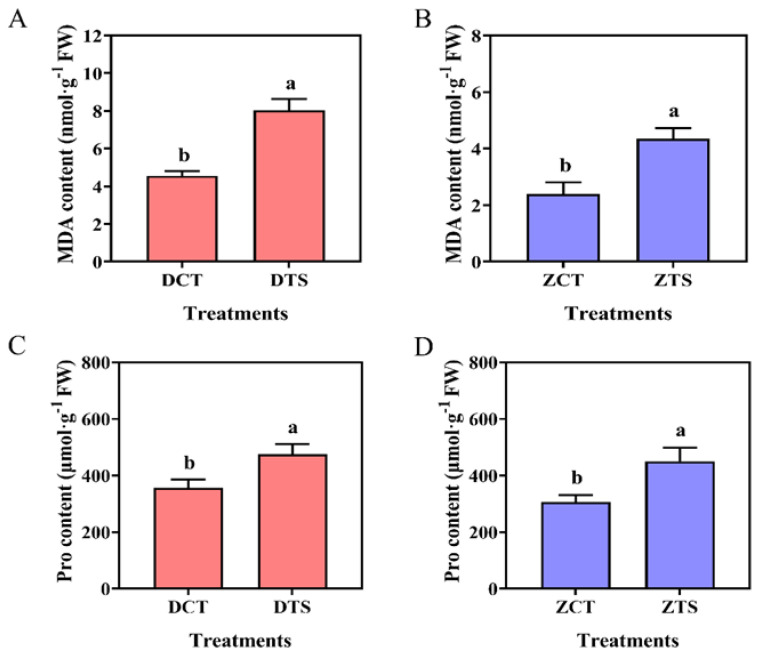
The effect of TS on the physiological changes iin the radicles in maize seeds. (**A**,**B**) show the changes in MDA content in the radicles of DMY1 and ZD958 germinated seeds after incubation under CT or TS conditions for 48 h, respectively; (**C**,**D**) show the changes in Pro content in the radicles of DMY1 and ZD958 germinated seeds after incubation under CT or TS conditions for 48 h, respectively. CT: control; DCT: control treatment of DMY1; DTS: low-temperature treatment of DMY1; ZCT: control treatment of ZD958; ZTS: low-temperature treatment of ZD958; MDA: malondialdehyde; Pro: proline. Data represent the mean ± standard error of four replicates. Means with different letters indicate significant differences between the different treatments (*p* < 0.05, Duncan’s multiple range test).

Malondialdehyde is a well-studied product of oxidative stress-induced lipid peroxidation which is also an indirect indicator of ROS levels [47]. TS treatments resulted in a rapid increase in MDA content in DMY1 and ZD958 radicles. The magnitude of change in MDA content in DMY1 radicles was greater than that in ZD958 after TS compared to normal culture conditions (Figure 2A,B). Pro content in DMY1 and ZD958 radicles increased by 33.64% and 46.96%, respectively, after 48 h of TS compared with the normal culture conditions (Figure 2C,D).

### 3.2. RNA Sequencing, Quality Control Analysis, and qPCR Validation

To gain insight into the in vivo molecular mechanisms occurring in the radicle during maize germination under TS conditions, the total RNA of radicles from different maize varieties under CT and TS treatments were sequenced using the high-throughput sequencing platform Illumina Hiseq. A total of 12 samples obtained 90.71 Gb of clean data, with 6.02 Gb of clean data per sample. The Q30 base percentages of all sequenced samples in this study were 89.42% or higher, while the GC content was approximately 53%, indicating that the sequencing data were reliable (Table S2). All sequencing data were uploaded to the NCBI database (login number: PRJNA1050059) (Table S3).

**Figure 3:**
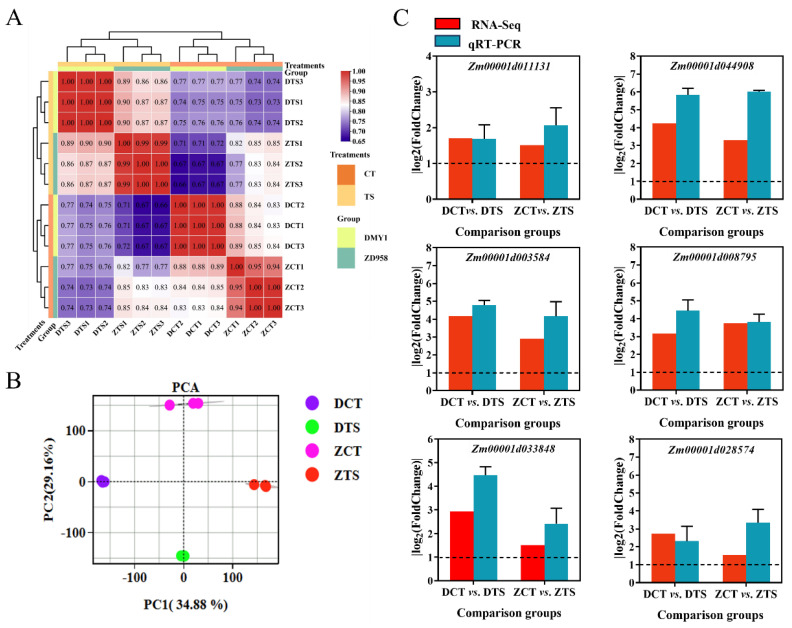
Expression and qPCR validation analysis of transcripts in maize radicles under TS. (**A**) Hierarchical clustering of transcript expression based on each maize radicle sample and correlation of expression per two samples. (**B**) Principal component analysis (PCA) showing the similarity of transcriptome samples across treatment conditions. (**C**) Quantitative RT-PCR used to verify the expression levels of six genes selected at random relative to each other in DCT vs. DTS and ZCT vs. ZTS. Red bars indicate RNA-seq data (|log_2_FoldChange|), cyan bars indicate qPCR data (|log_2_2^−ΔΔCt^|). DCT: control treatment of DMY1; DTS: low-temperature treatment of DMY1; ZCT: control treatment of ZD958; ZTS: low-temperature treatment of ZD958.

Samples from both maize varieties tended to cluster under CT and TS treatments (Figure 3A). Principal component analysis (PCA) of the transcriptome sequencing data showed high concordance between the three biological replicates in the four treatments of the 12 samples. Both varieties showed significant divergence from the control treatment under TS treatment, indicating that TS induced specific differential expression of genes in the maize radicle and resulted in some variation in the levels of gene expression under TS conditions between the two maize varieties (Figure 3B). Additionally, six randomly selected DEGs were selected and analysed by qPCR to verify RNA-seq data accuracy (Table S1). Quantitative PCR analysis showed that the two comparison groups of DCT vs. DTS and ZCT vs. ZTS with six DEGs had relative expression trends like those of transcriptome sequencing, demonstrating the dependability of transcriptome sequencing findings (Figure 3C).

### 3.3. Identification of DEGs and Enrichment Analysis of GO and KEGG

**Figure 4:**
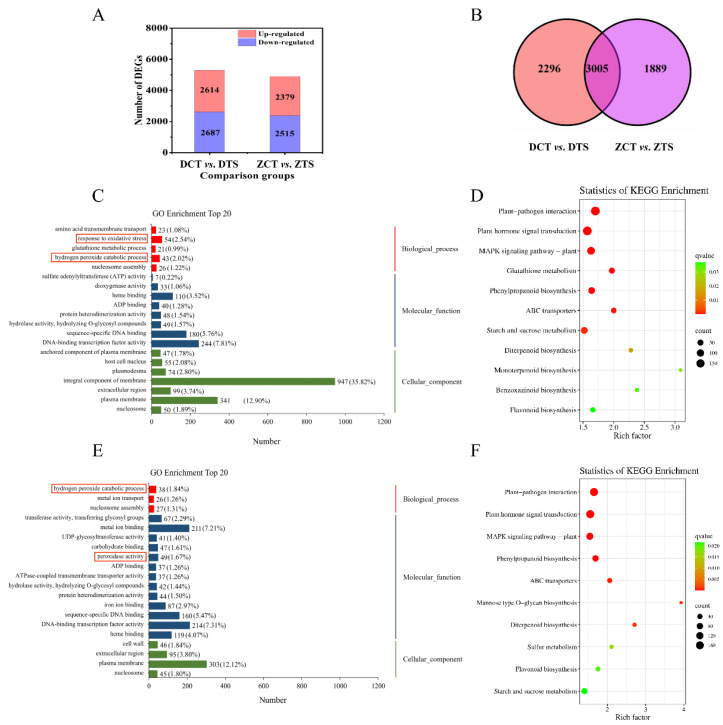
(**A**) Number of differentially expressed genes (DEGs) in comparison groups DCT vs. DTS and ZCT vs. ZTS. (**B**) Venn diagrams depict the differentially expressed and co-expressed DEGs in response to low temperature in DMY1 and ZD958 radicles. (**C**) Top 20 GO term analysis for the DCT vs. DTS comparison group. (**E**) Top 20 GO term analysis for the ZCT vs. ZTS comparison group. There are three main categories of Gene Ontology (GO) terms: biological process, molecular function, and cellular component. (**D**) The Kyoto Encyclopaedia of Genes and Genomes (KEGG) pathway for comparison group DCT vs. DTS. (**F**) KEGG pathway for comparison group ZCT vs. ZTS. DCT: control treatment of DMY1; DTS: low-temperature treatment of DMY1; ZCT: control treatment of ZD958; ZTS: low-temperature treatment of ZD958.

To compare the transcriptome differences in maize radicles under different treatments (CT and TS), we constructed two comparison groups: DCT vs. DTS and ZCT vs. ZTS. In total, 5301 DEGs were identified in DCT vs. DTS, of which 2614 were upregulated and 2687 were downregulated. Similarly, 4894 DEGs were identified in ZCT vs. ZTS, of which 2379 were upregulated and 2515 were downregulated (Figure 4A). In total, 3005 DEGs were co-expressed between DCT vs. DTS and ZCT vs. ZTS (Figure 4B, Table S4). The comparison of DCT vs. DTS revealed a greater number of DEGs than ZCT vs. ZTS.

GO enrichment analysis was performed to examine the function of the screened DEGs. DCT vs. DTS and ZCT vs. ZTS were significantly enriched (*q* < 0.05) with 62 and 38 GO terms, respectively; DEGs in comparison group DCT vs. DTS were mainly enriched in response to oxidative stress (GO: 0006979) and hydrogen peroxide catabolic processes (GO: 0042744), among others; whereas DEGs in ZCT vs. ZTS were mainly enriched to hydrogen peroxide catabolic process (GO: 0042744), peroxidase activity (GO. 0004601), and other GO terms (Figure 4C,E, Table S5). GO enrichment results showed that both maize varieties were enriched with two antioxidant-related GO terms to reduce the adverse effects of TS.

KEGG pathway enrichment was performed to understand these DEGs’ biological functions. In comparison groups DCT vs. DTS and ZCT vs. ZTS, 11 and 10 regulatory pathways were significantly enriched, respectively (Figure 4E,F, Table S6). Among them, plant–pathogen interaction, MAPK signaling pathway–plant, plant hormone signal transduction, phenylpropanoid biosynthesis, ABC transporters, starch and sucrose metabolism, diterpenoid biosynthesis, and flavonoid biosynthesis pathways were significantly enriched in both comparison groups (Figure 4D,F). Based on the *q*-value, plant–pathogen interaction, plant hormone signal transduction, MAPK signalling pathway–plant, and phenylpropanoid biosynthesis pathways were selected for the next step of this study.

### 3.4. Effect of TS on DEGs in Different Metabolic Pathways in Maize Radicles

**Figure 5:**
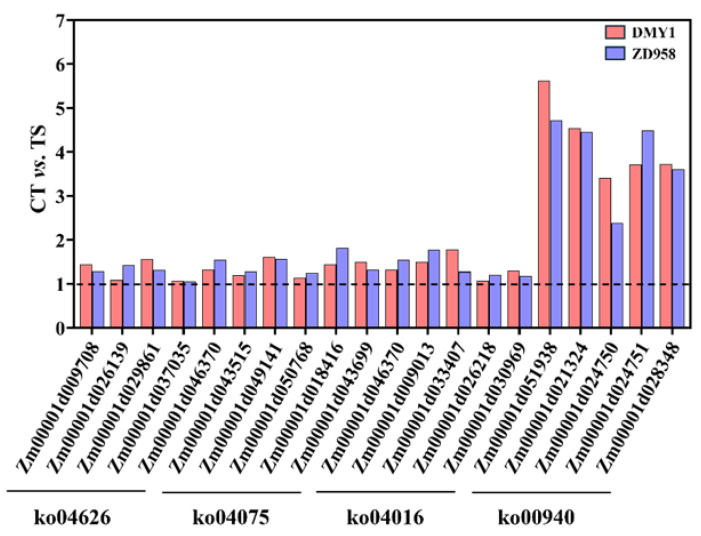
Expression comparison of some differential genes in plant–pathogen interaction, plant hormone signal transduction, MAPK signaling pathway–plant, and phenylpropanoid biosynthesis pathways in radicles of DMY1 and ZD958 maize under TS. ko04626: plant–pathogen interaction; ko04075: plant hormone signal transduction; ko04016: MAPK signalling pathway–plant; ko00940: phenylpropanoid biosynthesis. CT: control treatment; TS: low-temperature stress.

Five DEGs were randomly selected from each of the four metabolic pathways, plant–pathogen interaction (ko04626), plant hormone signal transduction (ko04075), MAPK signaling pathway–plant (ko04016), and phenylpropanoid biosynthesis (ko00940), and analysed (Figure 5). The |log_2_FC| values of the 20 selected DEGs were all >1, indicating that all 20 genes underwent significant expression changes at the transcriptional level under TS conditions. Among them, the phenylpropanoid biosynthesis pathway showed higher fold expression of DEGs, revealing the phenylpropanoid biosynthesis pathway as a potential candidate pathway for maize radicles to respond to TS.

### 3.5. Analysis of Phenylpropanoid Biosynthesis Pathways in Maize Radicles under TS

**Figure 6:**
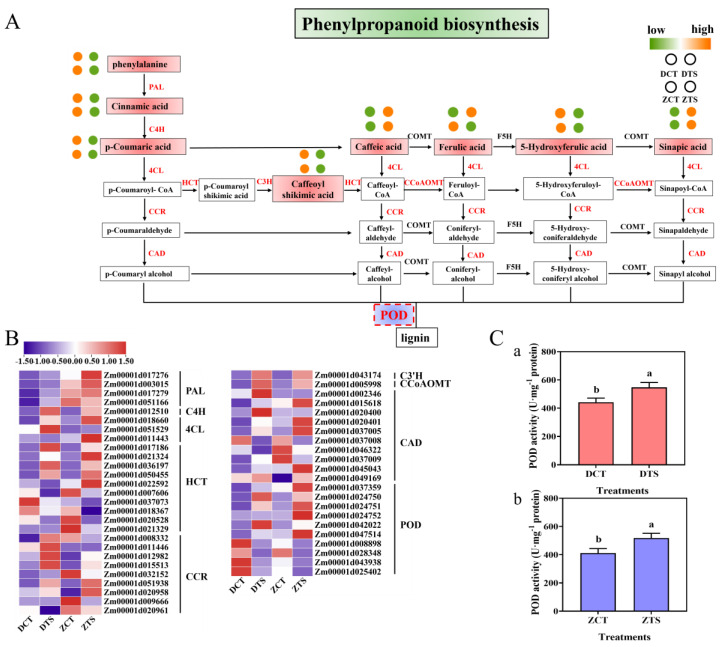
Analysis of phenylpropanoid biosynthesis pathways and determination of peroxidase (POD) enzyme activity in maize radicles of DMY1 and ZD958 under TS. (**A**) Metabolic changes in DMY1 and ZD958 under TS. (**B**) Transcriptional changes in DMY1 and ZD958 genes under TS. (**C**) Changes in POD activity in the radicles of DMY1 (a) and ZD958 (b) germinated seeds after incubation under CT or TS conditions for 48 h, respectively. DCT: control treatment of DMY1; DTS: low-temperature treatment of DMY1; ZCT: control treatment of ZD958; ZTS: low-temperature treatment of ZD958.

A total of nine key enzymes encoded by DEGs and eight different metabolites were identified from the phenylpropanoid biosynthesis pathway in radicles of DMY1 and ZD958 maize varieties in response to TS (Figure 6A,B, Table S4). Among them, phenylalanine, *p*-coumaric acid, cinnamic acid, 5-hydroxyferulic acid, and 5-O-caffeoylshikimic acid in radicles of both varieties showed a downregulated trend change in expression in response to TS, while caffeic acid and sinapic acid showed an upregulated trend change in expression. However, ferulic acid showed an opposite trend in the changes of the two varieties of radicles against TS. Meanwhile, most of the DEGs related to the above metabolites showed some expression pattern differences in two varieties; for example, under TS, some of the genes encoding PAL, 4CL, cinnamoyl-CoA reductase (CCR), shikimate O-hydroxy cinnamoyl transferase, and cinnamyl-alcohol dehydrogenase were more highly expressed in ZD958 than in DMY. Meanwhile, the genes encoding C4H, caffeoyl-CoA O-methyltransferase (CCoAOMT), and 5-O-(4-coumaroyl)-D-quinate 3′-monooxygenase (C3H) were expressed at a higher level than that of ZD958 in DMY1. Furthermore, seven key genes encoding POD had a fold change in expression of four-fold or more in both varieties, with three genes upregulated in expression and four genes downregulated in expression. TS significantly induced an increase in POD activity (Figure 6C). Compared with normal culture conditions, POD activities in DMY1 and ZD958 radicles increased by 24.01% and 25.77%, respectively, after 48 h of TS, which is a good indication that changes in DEGs encoding POD and enzyme activities in the phenylpropanoid biosynthesis pathway probably promoted maize radicle tolerance to TS. In conclusion, TS induces changes in the content of secondary metabolites through regulating genes expression in the phenylpropanoid biosynthesis pathway, and these secondary metabolism changes may be significant in improving the response and adaptation of maize radicles to low temperatures.

### 3.6. Homologous Sequence Comparison of Phenylpropanoid Biosynthesis Pathway Genes

**Figure 7:**
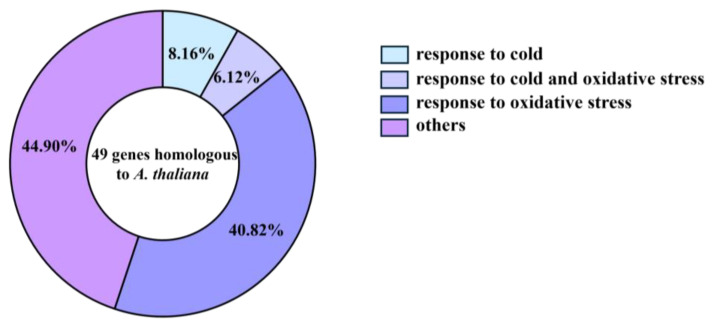
Homologous sequence comparison of phenylpropanoid biosynthesis pathway differential genes in maize radicles under TS in *Arabidopsis thaliana*.

Comparing DEGs in phenylpropanoid biosynthesis pathways to the *Arabidopsis thaliana* genome matched 49 *A. thaliana* homologs (Table S7). Some of these 49 *A. thaliana* genes participate in the cold response, and nearly half of them are involved in the oxidative stress response, according to a previous report (Figure 7, Table S7). Further explanation suggests that approximately 60% of the DEGs found in this study are related to cold tolerance in the phenylpropanoid biosynthesis pathway. Among them, genes *AT1G05260* (*Zm00001d024750*, *Zm00001d024751*, and *Zm00001d024752*) and a homologous *A. thaliana* gene encoding POD-related genes are involved in cold response and oxidative stress response [48,49]. They may be important candidate genes for promoting low-temperature resistance in maize radicles at the germination stage. This suggests that POD may be an important indicator for assessing cold tolerance in maize during germination.

## 4. Discussion

The process of seed germination and the subsequent growth of seedlings is greatly influenced by the environment; in particular, spring-sown maize is vulnerable to low temperatures. The germination of maize seeds is delayed and the normal growth of seedlings is hindered under low temperatures. Ultimately, this damage can lead to yield reductions in individual maize plants and entire populations [50,51,52]. The radicle is a nutritive organ in plants that absorbs water and nutrients. Germination success and population yield are strongly correlated with the normal breakdown of the seed coat and elongation of the radicle during germination. A study of 222 inbred maize lines under low-temperature conditions found large phenotypic differences in radicle length and a strong correlation between radicle length and germination rate [53]. Hence, the ability of maize seed radicles to withstand TS and achieve a high germination rate is vital for the overall growth, development, and production of maize. Furthermore, TS can affect plant morphology and physiology [54]. TS increases proline (Pro) content in plants [55]. In this study, using the artificial climate chamber simulation method widely utilised by previous researchers [37,56], we found that TS significantly inhibited the growth of radicles in the two test maize varieties (DMY1 and ZD958), resulting in a significant reduction in radicle growth, FW, and DW (*p* < 0.05). Additionally, the Pro content was significantly higher (*p* < 0.05) in radicles of both maize varieties under TS than under normal culture conditions. Malondialdehyde content is an indicator that reflects the permeability of the membrane; it is used as a measure of the cold tolerance of the plant. Plants that are more severely damaged by low temperatures have a higher MDA content [57]. In our study, the MDA content of the two maize varieties subjected to TS was significantly higher than that of CT. Similar results were found in maize inbred varieties SM and RM, where TS lead to a significant increase in Pro and MDA content in maize seeds [58]. The results mentioned above also suggest that the maize radicle samples under TS in this study can be utilised for transcriptomic analysis to gain a molecular understanding of the response of maize radicles to TS during seed germination.

Transcriptome sequencing can be an important way to anchor candidate genes and pathways by mining plant resistance mechanisms in response to TS through gene expression. In DMY1 and ZD958 radicles, we identified 5301 and 4894 DEGs, respectively, of which 3005 DEGs were shared. Many cold-responsive genes were identified in maize in previous studies [59,60,61]; for example, 357 and 455 DEGs were identified in two sh2 inbred lines, RC and C5, respectively, and 94 of these genes were associated with cold response [62]. Excess ROS produced by plants in response to various types of stresses pose a danger for cells while triggering various defence mechanisms and pathways of stress [63]. The increase in POD activity removes excess ROS and free radicals from plants, thereby reducing damage to plant tissues from stressful environments [64,65]. In the existing study, some terms enriched to GO, such as response to oxidative stress (GO: 0006979), peroxidase activity (GO: 0004601), and hydrogen peroxide catabolic processes (GO: 0042744), are associated with plant stress tolerance [66,67]. In tobacco subjected to cold domestication, some DEGs and differential metabolites (which have regulatory roles in phenylpropanoid biosynthesis) are involved in low-temperature defence processes [68]. POD is an antioxidant enzyme closely related to phenylpropanoid biosynthesis [69]. The results of GO enrichment in this study showed that two GO terms related to antioxidant pathways were enriched in DEGs of DMY1 and ZD958 radicles under TS. KEGG analysis revealed that DEGs from radicles of two maize varieties in response to TS were mainly enriched in the phenylpropanoid biosynthesis pathway, indicating that the phenylpropanoid biosynthesis pathway plays significant roles in maize radicle response to TS, and that POD may be an important indicator for assessing the cold tolerance of maize radicles during the germination stage.

**Figure 8:**
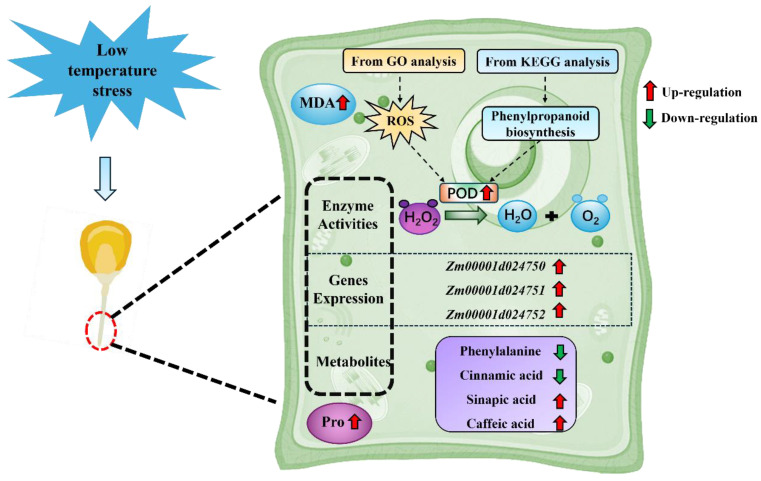
Schematic of the mechanism of phenylpropanoid biosynthesis under TS in the maize radicle. Red arrow represents upregulation, whereas green arrow represents downregulation.

TS leads to excessive accumulation of ROS in the cell membrane, resulting in cellular damage [70,71]. The phenylpropanoid biosynthesis pathway is recognised as one of the important pathways affecting plant secondary metabolite synthesis. It is activated under abiotic stress conditions, leading to accumulation of a variety of phenolic compounds [72,73]. These phenolic substances have strong antioxidant capacity and provide plants with oxidative stress protection against lipid peroxidation damage [74,75,76]. PAL and C4H are involved in the conversion of phenylalanine to several phenolic acids (such as trans-cinnamic acid, *p*-coumaric acid, caffeic acid, ferulic acid, and sinapic acid) [77,78]. Among them, ferulic, coumaric, and sinapic acids play important roles in plant defence responses [79,80,81]. It has been shown that TS results in reduced levels of caffeate, ferulic acid, and *p*-coumaraldehyde in poplars [82], and an increase in *p*-coumaryl alcohol, ferulic acid, caffeic acid, and sinapyl alcohol levels in *Camellia oleifera* [83]. In the present study, caffeic acid and sinapic acid contents showed an increasing trend in radicles of the two varieties under TS, but ferulic acid showed the opposite trend in resistance to TS, indicating that caffeic, sinapic, and ferulic acids have a similar positive response to the TS process in maize radicles, but the trends may vary by species. CCR, C3H, and CCoAOMT can enhance the mechanical strength of plants by regulating lignin biosynthesis, promoting water transport in tissues and resisting unfavourable environmental conditions [84,85]. The 4CL gene has a function in regulating resilience, and abiotic stresses lead to an increase in 4CL enzyme activity [86,87,88]. POD can determine the functions of cell wall lignification, cell elongation, stress defence, phytohormone regulation, and structural protein formation [89]. In this study, most of the genes encoding PAL, C4H, 4CL, CCR, C3H, and CCoAOMT were upregulated for expression under TS. Analysis of POD activity and gene expression levels of its genes encoding POD showed that expression of genes encoding POD was changed in the phenylpropanoid biosynthesis pathway under TSs such as *Zm00001d024750*, *Zm00001d024751*, and *Zm00001d024752*, and significantly elevated the POD activity (*p* < 0.05). This suggests that high or low POD activity and the expression levels of the encoding genes in maize radicles could be crucial factors in the future evaluation of the ability of the radicle to respond to TS and mitigate low-temperature injury. In summary, TS induced changes in key enzymes for phenolic substances in the maize radicle, which could effectively increase the phenolic substance content and enhance the plant’s stress tolerance (Figure 8). As the present study was a seed germination experiment based on the paper-bed method in an artificial climate chamber, it is imperative to verify the above regulatory mechanisms of the radicle response to low-temperature stress in different maize varieties under field conditions from sowing until seedling emergence in future experiments.

## 5. Conclusions

We selected two maize varieties (DMY1 and ZD958) that are mainly grown in regions of northeastern China with cold high-latitude climates and comprehensively compared and analysed the phenotypic, physiological, and transcriptomic differences in the maize radicle growth during germination under TS conditions. Overall, low temperatures inhibited maize radicle elongation, leading to a decrease in radicle fresh and dry weights, while increasing MDA and Pro content. Additionally, 5301 and 4894 DEGs were identified in DMY1 and ZD958, respectively, of which 3005 genes were significantly differentially expressed in both varieties, suggesting that DMY1 and ZD958 have many common and unique molecular mechanisms involved in seed germination tolerance to TS. Analysis of DEGs revealed that the phenylpropanoid biosynthesis pathway is a potentially important candidate pathway involved in TS. Studies of metabolite expression and POD activity further indicated that the phenylpropanoid biosynthesis pathway is actively involved in the maize radicle response process to TS, that POD may be a key indicator for assessing low-temperature stress response in the future, and that the key genes encoding POD, such as *Zm00001d024750*, *Zm00001d024751*, and *Zm00001d024752*, could serve as important candidate genes for exploring the mechanism of low-temperature response during maize germination. This study deepens the understanding of the molecular physiological mechanisms of radicle adaptation during maize seed germination to TS and provides a theoretical basis for efficiently assessing maize seed low-temperature tolerance and improving maize adversity germination performance in the future.

## Data Availability

Data are contained within the article and Supplementary Materials.

## Supplementary Materials

The following supporting information can be downloaded at: https://www.mdpi.com/article/10.3390/plants13101362/s1, Table S1: Primers for qPCR validation; Table S2: Sequencing quality control. Table S3: Raw data of transcriptome uploaded to the NCBI database. Table S4: DEGs in DMY1 and ZD958. Table S5: GO enrichment analysis of two comparison groups. Table S6: KEGG enrichment analysis of the two comparison groups. Table S7: Homologous sequence alignment of phenylpropanoid biosynthesis pathway genes in maize radicles under low-temperature stress.
